# The Mediating Role of Family Communication in the Relationship Between Academic Achievement Pressure and Problematic Smartphone Use Among Korean Early Adolescents

**DOI:** 10.3390/children12091141

**Published:** 2025-08-28

**Authors:** Hwa-Mi Yang

**Affiliations:** Department of Nursing, Daejin University, Pocheon 11159, Republic of Korea; yhm2021@daejin.ac.kr; Tel.: +82-31-539-1878

**Keywords:** adolescents, behavior, addictive, children, communication, mediation analysis, smartphone

## Abstract

**Highlights:**

**What are the main findings?**

- Higher perceived parental academic achievement pressure is associated with an increased risk of problematic smartphone use in early adolescence.
- Family communication significantly mediates the relationship between academic pressure and smartphone addiction risk.

**What is the implication of the main finding?**

- Improving family communication may help buffer the negative effects of parental academic pressure on adolescent behavior.
- Family-focused interventions could be effective in preventing problematic smartphone use among adolescents under high academic stress.

**Abstract:**

**Background/Objectives:** Problematic smartphone use among early adolescents is a growing public health concern, often influenced by psychosocial stressors such as parental academic achievement pressure. Despite evidence linking academic pressure to adolescent stress and maladaptive coping behaviors, the mediating role of family communication in this relationship remains underexplored. This study investigates whether family communication mediates the association between perceived parental academic pressure and problematic smartphone use in early adolescents. **Methods**: Using a cross-sectional correlational design, data from the 15th wave (2022) of the nationally representative Public Korean Children’s Panel (PSKC) were analyzed, including 1249 adolescents born in 2008. Logistic regression and mediation analyses assessed direct and indirect relationships among variables. **Results**: Among participants, 16.2% were classified as potential or high-risk smartphone users. Higher perceived parental academic pressure significantly increased the odds of problematic smartphone use (OR = 1.44, 95% CI: 1.20–1.72), whereas better family communication was associated with lower odds (OR = 0.66, 95% CI: 0.53–0.83). Mediation analysis showed that family communication partially mediated the effect of academic pressure on smartphone addiction. **Conclusions**: These findings highlight family communication as a key psychosocial mechanism buffering the negative impact of parental academic pressure on adolescent smartphone use. Enhancing family communication may be a critical focus for interventions to prevent problematic smartphone behaviors in adolescents facing high academic demands.

## 1. Introduction

In modern society, smartphones have become indispensable tools for daily life, fulfilling various roles including information acquisition, communication, and leisure activities. Adolescents today represent the first generation to have grown up fully immersed in an environment saturated with smartphones and internet access, leading them to adopt these technologies more rapidly and use them more extensively than adults [1]. This extensive use of smartphones has resulted in both positive and negative outcomes. Several studies have shown that while smartphone use can enhance social connectivity and help build relationships [2], it may also contribute to higher levels of anxiety, stress, and social isolation among adolescents [3].

Despite their social connectivity benefits, excessive smartphone use among adolescents has been associated with several adverse outcomes, including depressive symptoms, sleep disturbances, and diminished academic performance [1]. Although excessive smartphone use is not officially classified as a disorder in the DSM–5 or ICD–10, it shares many traits with other behavioral addictions [4]. Therefore, problematic smartphone use is considered a compulsive behavior that leads to negative physical, psychological, or social consequences [4].

A meta-analysis by Yang et al. (2020) confirms these associations, emphasizing the significant correlation between excessive smartphone use and mental health issues such as depression and anxiety [5]. As the global prevalence of smartphone use increases, smartphone-related problems are emerging as a significant public health concern worldwide. The widespread availability of mobile devices and continuous internet access have made constant connectivity a part of daily life. With over 8.89 billion mobile subscriptions and 5.4 billion internet users globally, this pervasive reliance on smartphones has contributed to the rising issue of smartphone addiction across countries [6].

The global prevalence of problematic smartphone use was estimated at 37.1% from 2012 to 2022, and has steadily increased over time [7]. In line with this global trend, the 2024 Korea Youth Survey found that 42.6% of adolescents are at risk for smartphone use problems [8]. Adolescence is a crucial developmental period for forming self–identity and social relationships, and smartphone addiction during this stage can negatively impact an individual’s mental health and quality of life [9]. In particular, family factors, including family environment, expectations, and parental attachment, play a critical role in adolescents’ problematic smartphone use [10,11]. A comprehensive understanding of these factors is essential for developing effective strategies to mitigate problematic smartphone use during adolescence

One of the associated factors of smartphone addiction is parental pressure regarding academic achievement. In modern society, parents intervene in various ways to ensure their children’s success and adjustment, with one of the primary methods being the expectation of high achievement. This pressure is firm in East Asian countries like Korea, where academic performance is often tied to social and familial status [12]. Particularly among Korean adolescents, parental high academic expectations and intense achievement pressure are known to place excessive psychological burdens on children, negatively impacting their psychological well-being [13]. Academic pressure may lead adolescents to use smartphones excessively as a coping mechanism to escape negative emotions. According to Flynn, Thériault, and Williams (2020), this dependency, in turn, is associated with avoidance and emotion-focused coping strategies, reinforcing the cycle of excessive smartphone use as a means of emotional escape [14].

When parents convey the pressure to achieve to their children in a critical and controlling manner, the children may perceive this as overwhelming pressure and stress. This type of communication can lead to a loss of self-confidence or anxiety when the children do not meet the parents’ expectations. Children may become addicted to their cell phones to relieve stress from academic pressure, and the negative consequences of academic stress can also lead to depression [15]. Adolescents experience significant academic pressures that demand effective coping skills, which are often relieved by social resources like parental support and family interactions [16]. Adolescents who receive more parental support tend to adopt adaptive coping strategies, such as problem-solving or seeking comfort. In contrast, those with more negative interactions with their parents are more likely to engage in avoidance behaviors like concealment or escape [16]. These findings highlight that the ability to cope with academic stress is closely tied to the quality of parent-adolescent relationships, even during the teenage years.

In this context, the communication patterns within the family serve as an important environmental factor that can moderate or mediate the impact of parental expectations on children. Family communication theory explains how communication patterns among family members affect the family’s functioning and each member’s psychological and emotional health. In this theory, communication is viewed not merely as the transmission of information but as a crucial social process that includes emotional support, the quality of relationships, and conflict resolution strategies [17]. In families with open and supportive communication, children are more likely to embrace parental expectations without feeling overwhelmed and respond positively. Conversely, in families with poor or negative communication, children may experience psychological stress and emotional isolation, which can lead to avoidant behaviors, such as excessive smartphone use. Furthermore, parents often fail to recognize the underlying psychosocial difficulties that drive their children to use problematic smartphones [18]. One key reason for this is that many children struggle to effectively communicate their emotional challenges, which makes it difficult for parents to identify and understand these issues [18].

Recent studies have explained the relationship between parental academic pressure and adolescent addictive behaviors through various psychological pathways. Kaymakcı, Ekşi [19] reported that parental academic pressure directly influences adolescents’ gaming addiction, with emotional regulation difficulties playing a mediating role in this relationship. Yaghoobi, Karimi [20] found that academic stress affects smartphone addiction, with the psychological resource of ‘wisdom’ mediating this relationship. Additionally, Yunjie, Chongyong [21] suggested that academic stress can lead to mobile gaming addiction through a decrease in self-control. However, there remains a lack of research that integrates the role of family communication patterns into these psychological pathways, which this study aims to address. While much of the existing literature has focused on the psychological traits that contribute to smartphone addiction, recent studies have emphasized the significance of family dynamics in shaping adolescent behavior [10,11]. However, research on how parental expectations are communicated through family communication, particularly concerning academic achievement pressure, remains limited. This study aims to address this gap by exploring the mediating role of family communication in the relationship between academic pressure and problematic smartphone use among Korean early adolescents. Accordingly, this study tested the following hypotheses.

**Hypothesis** **1.** Parental academic pressure is positively associated with adolescents’ problematic smartphone use.

**Hypothesis** **2.** Parental academic pressure is negatively associated with family communication.

**Hypothesis** **3.** Family communication is negatively associated with adolescents’ problematic smartphone use, controlling for parental academic pressure.

**Hypothesis** **4.** Family communication mediates the relationship between parental academic pressure and adolescents’ problematic smartphone use.

## 2. Materials and Methods

### 2.1. Study Design

This cross-sectional correlational study aimed to examine the relationship between academic achievement pressure and smartphone addiction in early adolescents in Korea, with family communication as a mediating variable. The study was conducted with 1249 Korean early adolescent participants.

### 2.2. Data and Study Participants

The data used in this study were drawn from the Panel Study on Korean Children (PSKC), a large-scale, nationwide cohort study conducted by the Korea Institute of Childcare and Educational Research [22]. For sampling, the PSKC employed a stratified multistage sampling methodology [23]. In this process, the population was first stratified based on geographic region and residential area type (urban vs. rural) [23]. This stratification ensured that the sample was representative of various regions across Korea, including both urban and rural areas. Stratifying the sample in this manner was crucial for capturing a broad range of socio-economic and environmental factors that may influence children’s development, providing a more comprehensive picture of the national population. The PSKC is a longitudinal study that tracks a representative sample of parents and their children, all of whom were born between April and July 2008 [22]. This dataset is publicly accessible and available to researchers for academic use. The first wave of data collection occurred in 2008, with the 15th wave conducted in 2022. The primary objective of the survey is to investigate various aspects of children’s growth and development, parenting practices, and the impact of childcare policies and support systems [24].

This study utilized data from the 15th wave (2022) of the Public Korean Children’s Panel (PSKC), focusing on 1249 early adolescents who participated in the survey that year. Out of the total 2150 respondents in the PSKC, 856 individuals were excluded due to missing responses on the key variable, academic achievement pressure; 1 participant was excluded for not reporting gender; and 44 participants were excluded for not responding to the problematic smartphone use items. After these exclusions, the final sample consisted of 1249 participants.

### 2.3. Measurements

Subjective health status and perceived socioeconomic status were measured as part of the demographic and socioeconomic characteristics. Subjective health status was assessed through parental reports using the question “How would you assess your child’s health status?” rated on a 5-point Likert scale from 1 (very unhealthy) to 5 (very healthy), with higher scores indicating better perceived health. Perceived socioeconomic status was measured by asking adolescents to indicate their family’s economic position on a 10-point scale ranging from 1 (very low socioeconomic status) to 10 (very high socioeconomic status), where higher scores reflect a higher perceived socioeconomic standing.

#### 2.3.1. Parental Academic Achievement Pressure

Parental academic achievement pressure, as perceived by children, refers to the pressure exerted by parents who demand and expect high academic performance from their children. The instrument used in this study was developed based on selected items related to achievement pressure from Kim Ki-jung’s (1984) Parental Child-rearing Attitude Scale and the parenting behavior subscale of academic achievement pressure from Kim Kyung-ok’s (1992) work, as adapted and finalized by the Korea Institute of Child Care and Education for the Public Korean Children’s Panel. The final scale consists of 15 items. Each item is rated on a 5-point Likert scale ranging from 1 (strongly disagree) to 5 (strongly agree), with higher scores indicating greater perceived parental academic pressure as reported by adolescents. The average score of all items was used in the analysis. Example items include “My parents say that to become a successful person, I must study hard,” “My parents worry that I spend less time studying because of my friends,” “My parents scold me when my school grades drop slightly,” and “My parents tell me that I must achieve better grades than others.” Han and Park (2024) and Kim (2025) reported a Cronbach’s alpha of 0.83 for this scale [25,26]. In the current study, Cronbach’s alpha was 0.94.

#### 2.3.2. Family Communication

Family communication was assessed using a Korean-adapted version of the Family Communication Scale from the Family Adaptability and Cohesion Evaluation Scales IV (FACES IV), initially developed by Barnes and Olson (1985) and later revised by Olson (2011). This Korean version, adapted by Kim Young-sik et al. (2012) to reflect cultural nuances, was employed in the current study through data from the Public Korean Children’s Panel. The scale comprises 10 items, each rated on a 5-point Likert scale ranging from 1 (strongly disagree) to 5 (strongly agree), with higher scores indicating more effective communication between parents and their adolescent children. Sample items include “My family listens well to each other,” “My family expresses affection to one another,” and “When my family asks questions, honest answers are given.” Yuh (2025) reported a Cronbach’s alpha of 0.93 for this scale [27], and the present study also found a Cronbach’s alpha of 0.93, demonstrating high internal consistency.

#### 2.3.3. Problematic Smartphone Use

Problematic smartphone use refers to excessive smartphone usage that negatively impacts daily life, academic performance, and interpersonal relationships. The problematic smartphone use scale employed in this study was developed in 2011 by the Korea Information Society Development Institute for adolescents. The scale consists of 15 items rated on a 4-point Likert scale ranging from 1 (strongly disagree) to 4 (strongly agree). An example item is “Excessive smartphone use has caused my school grades to decline.” Items 8, 10, and 13 (e.g., “I can control my smartphone usage time”) were reverse coded for analysis. Higher total scores indicate more severe problematic smartphone use.

The scale is divided into the following subfactors, with item sums used for classification:

- Factor 1 (Daily Life Disruption): sum of items 1, 5, 9, 12, and 13;
- Factor 3 (Withdrawal): sum of items 3, 7, 10, and 14;
- Factor 4 (Tolerance): sum of items 4, 8, 11, and 15.

High–risk users were defined as those scoring 45 or above on the total score, or 16 or above on Factor 1, 13 or above on Factor 3, or 14 or above on Factor 4. Potential-risk users were those with total scores between 42 and 44, or scores of 14 or above on Factor 1, 12 or above on Factor 3, or 13 or above on Factor 4. Participants classified as high-risk or potential-risk groups were collectively defined as exhibiting problematic smartphone use. Based on these criteria, high-risk and potential-risk groups were coded as 1, while the general user group was coded as 0 for analysis. Lee (2024) reported a Cronbach’s alpha of 0.84 for this scale [28]. In the current study, Cronbach’s alpha was 0.85.

### 2.4. Data Analysis

All statistical analyses were conducted using IBM SPSS Statistics for Windows, Version 23.0 (IBM Corp., Armonk, NY, USA). Descriptive statistics were used to summarize participants’ demographic characteristics. Logistic regression analyses were performed to examine the crude associations between parental academic achievement pressure, family communication, and problematic smartphone use among early adolescents.

Logistic regression analysis was performed using composite variables derived from multi-item constructs. For each construct (e.g., parental pressure for academic achievement, family communication), item scores were averaged to generate a composite score, which was then entered as an independent or mediating variable in the regression model.

For the dependent variable, problematic smartphone use, factor scores were summed for each subscale. Based on predefined scoring criteria, the total scores were dichotomized into either a “high-risk/potential-risk” group or a “general user” group. This dichotomized variable was used as the outcome variable in the multiple logistic regression analysis.

To test the mediating effect of family communication on the relationship between parental academic pressure and problematic smartphone use, a three-step regression analysis was conducted following the Baron and Kenny [29] procedure, with adolescents’ perceived socioeconomic status included as a covariate in all models.

In the first step, logistic regression was used to assess the direct effect of parental academic pressure on problematic smartphone use. In the second step, multiple linear regression was conducted to examine the association between parental academic pressure and family communication. In the third step, logistic regression was applied to investigate the simultaneous effects of parental academic pressure and family communication on problematic smartphone use. The mediation effect of family communication was evaluated by comparing the regression coefficients from the first and third steps to determine whether the association between parental academic pressure and problematic smartphone use decreased or became non-significant when family communication was included as a mediator. Additionally, the Sobel test was performed to assess the statistical significance of the mediating effect. A *p*-value less than 0.05 was considered statistically significant.

## 3. Results

### 3.1. General Characteristics of the Participants

The average age of the adolescents was 14.3 years, with boys comprising 51.1% of the sample. The average subjective health status was 4.0 out of 5 points, and the average perceived socioeconomic status was 6.2 out of 10 points. Parental academic achievement pressure had a mean score of 2.4 on a 5-point scale, while family communication averaged 3.9 out of 5 points. Regarding problematic smartphone use, 16.2% of participants were classified as potential or high-risk users (Table 1).

### 3.2. Unadjusted Associations with Problematic Smartphone Use in Early Adolescents

Age, sex, and subjective health status were not found to be significantly associated with problematic smartphone use. In contrast, higher perceived socioeconomic status was significantly associated with a reduced likelihood of problematic smartphone use (B = −0.20, OR = 0.82, 95% CI: 0.74–0.91). Parental academic achievement pressure was positively associated with problematic smartphone use, indicating that greater parental academic achievement pressure corresponded to higher odds of problematic use (B = 0.36, OR = 1.44, 95% CI: 1.20–1.72). Moreover, a high level of family communication was significantly related to lower odds of problematic smartphone use (B = −0.41, OR = 0.66, 95% CI: 0.53–0.83) (Table 2).

### 3.3. Family Communication as a Mediator Between Parental Academic Pressure and Problematic Smartphone Use in Early Adolescents

Parental academic achievement pressure was positively associated with problematic smartphone use among early adolescents in the first equation (AOR [95% CI] = 1.39 [1.16–1.67], *p* < 0.001) (Table 3). In the second equation, parental academic achievement pressure was negatively associated with family communication (β = −0.16, *p* < 0.001).

**Figure 1:**
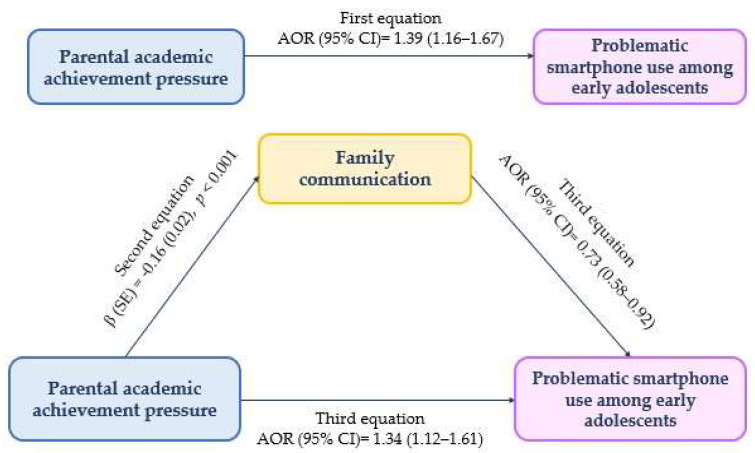
The mediating role of family communication on the link between parental academic achievement pressure and problematic smartphone use among early adolescents. β = unstandardized regression coefficient; CI = confidence interval; AOR = adjusted odds ratio; SE = standard error.

In the third equation, family communication remained significantly associated with problematic smartphone use, even after adjusting for parental academic achievement pressure. Although the strength of the association was slightly reduced compared to the first equation (AOR [95% CI] = 1.34 [1.12–1.61], *p* = 0.002), family communication continued to have a direct effect on problematic smartphone use (Z = −2.12, *p* = 0.03) (Table 3 and Figure 1).

## 4. Discussion

This study confirmed that family communication mediates parental academic achievement pressure and adolescents’ problematic smartphone use. These findings are significant in that excessive parental demands for academic achievement not only increase early adolescents’ psychological burden but also lead to a low level of family communication, which is associated with problematic smartphone use.

This study confirmed that parental academic pressure is positively associated with early adolescents’ problematic smartphone use. In support of this, Wang, Wang [30] reported that academic stress can lead to emotional problems such as depression, which in turn interact with various psychosocial factors, such as sleep deprivation, loneliness, and reduced physical activity, ultimately contributing to maladaptive behaviors like smartphone addiction. Similarly, Busari [31] also emphasized that academic stress can lead to internet addiction through the mediation of negative emotions. In particular, Jun and Choi [32], drawing on General Strain Theory, explained that when adolescents experience academic stress, it increases negative emotions such as anger and frustration, which may be expressed outwardly through addictive behaviors. In interpreting these findings, the stress-coping theory proposed by Lazarus and Folkman (1984) provides an essential theoretical framework. According to this theory, individuals cognitively appraise stressful situations, experience corresponding emotional responses, and choose appropriate coping strategies [33]. However, excessive academic pressure from parents increases psychological burdens and negative emotions in adolescents, which may lead them to adopt inefficient or maladaptive coping strategies. The absence of effective coping mechanisms can ultimately lead to problematic behaviors, such as excessive smartphone use. Therefore, the findings of this study suggest that psychological stress caused by academic pressure influences the emotional environment within the family and the coping styles employed by adolescents, each of which plays a significant role in the development of problematic smartphone use.

High parental academic expectations can create tension in the parent-child relationship and diminish the quality of family communication. Family Systems Theory conceptualizes the family as an interconnected system, wherein the behaviors of individual members are circular and mutually influential [34]. From this perspective, parental academic pressure may disrupt the emotional equilibrium of the family system, potentially leading to breakdowns in communication pathways. Supporting this view, Hari [35] found that excessive parental expectations can undermine adolescents’ self-esteem and exacerbate test anxiety, which in turn may contribute to negative interactions within the family. These previous studies suggest that the level of parental pressure is not only related to academic achievement but also has meaningful implications for family communication patterns and adolescents’ psychological well-being.

Family communication is associated with adolescents’ problematic smartphone use. Ulusoy and Atar [36] reported that lower levels of family communication are linked to a higher likelihood of adolescents relying on social media or smartphones. According to stress and coping theory, when family communication is weak, adolescents may perceive reduced emotional and social support from their families, which can hinder effective stress coping and increase vulnerability to maladaptive strategies such as excessive smartphone use. This behavior can be understood as defensive coping, in which adolescents turn to virtual environments for surrogate emotional satisfaction and a sense of belonging that they fail to receive within the family. Nikdel and Nasab [37] reported that family communication is associated with the fulfillment of basic psychological needs, and that higher satisfaction of these needs is linked to lower levels of internet addiction. In addition, Jabeen, Sarvat [38] and Monteiro, Simões [39] also confirmed a negative correlation between family communication and internet addiction. Egunjobi [40] emphasized that family communication plays a central role in addressing addiction problems, arguing that effective communication within the family is a crucial factor in both the prevention and recovery of addiction. Thus, stable communication and emotional exchange within the family are important environmental factors influencing adolescents’ excessive smartphone use.

This study empirically confirmed that family communication plays a significant mediating role in the impact of parental academic pressure on adolescents’ smartphone addiction. Tajalli and Zarnaghash [41] explained that parents’ conversation-oriented communication enhances adolescents’ mental health and self-efficacy, thereby helping to prevent internet addiction. These findings support the idea that when parents offer emotional support through open and trust-based communication with their children, it can effectively mitigate or prevent problematic smartphone use. Quach, Epstein [42] found that fathers’ academic pressure can exacerbate adolescents’ anxiety and depressive symptoms, while warmth serves as a buffer against these negative emotions. Luo and Zhang [43] explained that excessive parental pressure can stimulate adolescents’ extrinsic motivation and lead to negative behaviors, whereas appropriate expectations and emotional support strengthen intrinsic motivation and promote positive behaviors. These previous studies emphasize that parents should not focus solely on academic achievement in their relationship with their children, but also provide emotional support and warm interactions. This highlights the complex and profound influence that the quality of communication among family members has on adolescents’ health behaviors. Accordingly, this study empirically demonstrated that parental academic pressure can be negatively associated with adolescents’ smartphone addiction, with family communication serving as a key psychosocial mediating variable in this process. These findings highlight the need for a comprehensive family-centered approach to prevent and address problematic smartphone use among adolescents.

This study has several limitations. First, our analysis is based on South Korean adolescents, which may limit the generalizability of the findings to other countries with different cultural and societal contexts. Additionally, the study’s cross-sectional design restricts the ability to establish causal relationships. While our study highlights family communication as a mediator, it does not consider other potential factors, such as peer influence, genetic factors, individual personality traits, or societal expectations. In particular, future research may need to explore the possibility of uncovering genetic factors in smartphone and internet addiction, which could provide a deeper understanding of individual vulnerabilities.

## 5. Conclusions

This study confirmed that parental academic achievement pressure is negatively associated with early adolescents’ problematic smartphone use, with family communication significantly mediating this relationship. These findings suggest that adolescents’ smartphone addiction is not merely an issue of individual self-regulation but is closely linked to the psychosocial dynamics within the family. This supports prior research emphasizing the role of family functioning in adolescents’ digital media behaviors [44,45].

Therefore, rather than a unidirectional parenting approach, a focus on academic achievement, emotional support, and open communication should be emphasized. Such a relational approach aligns with family system theory, which posits that family interaction patterns influence children’s behavioral outcomes [46].

By fostering a supportive emotional environment at home, parents can play a crucial role in promoting adolescents’ emotional stability and encouraging healthier media use habits. These insights underscore the importance of incorporating family-based strategies into interventions designed to reduce problematic smartphone use among youth.

## Figures and Tables

**Table 1:** The characteristics of the participants (N = 1249).

Variables	N (%)	Mean ± SD	Range
Age		14.3 ± 0.14	14.0–14.8
Sex			
Boy	638 (51.1)		
Girls	611 (48.9)		
Self-rated health		4.0 ± 0.63	2.0–5.0
Perceived economic status		6.2 ± 1.46	1.0–10.0
Parental academic achievement pressure		2.4 ± 0.84	1.0–5.0
Family communication		3.9 ± 0.67	1.0–5.0
Problematic smartphone use			
High or potential risk group	202 (16.2)		
General user group	1047 (83.8)		

**Table 2:** Crude logistic regression relevant to problematic smartphone use among early adolescents (N = 1249).

Variables	β (SE)	OR (95% CI)	*p*-Value
Age	−0.16 (0.543)	0.85 (0.29–2.47)	0.852
Sex (Boy)	0.12 (0.154)	1.12 (0.83–1.52)	0.459
Self-rated health	−0.09 (0.121)	0.91 (0.72–1.15)	0.440
Perceived economic status	−0.20 (0.054)	0.82 (0.74–0.91)	<0.001
Parental academic achievement pressure	0.36 (0.091)	1.44 (1.20–1.72)	<0.001
Family communication	−0.41 (0.113)	0.66 (0.53–0.83)	<0.001

β = unstandardized regression coefficient; CI = confidence interval; OR = odds ratio; SE = standard error.

**Table 3:** Hypothesis testing for a mediating effect of family communication on the association between academic achievement pressure and problematic smartphone use among early adolescents (N = 1249).

Variables	β (SE)	AOR (95% CI)	*p*-Value
First equation Outcome variable: Problematic smartphone use Independent variable: Academic achievement pressure	0.33 (0.09)	1.39 (1.16–1.67)	<0.001
Second equation Outcome variable: Family communication Independent variable: Academic achievement pressure	−0.16 (0.02)		<0.001
Third equation Outcome variable: Problematic smartphone use Mediator: Family communication Independent variable: Academic achievement pressure * Sobel’s test, z = −2.12, *p* = 0.03	0.29 (0.09)	1.34 (1.12–1.61)	0.002

β = unstandardized regression coefficient; AOR = adjusted odds ratio; CI = confidence interval; SE = standard error. All multiple regression models were adjusted for perceived economic status; * Sobel’s test: indicates that the result was tested using Sobel’s test for mediation.

## Data Availability

The data used in this study were obtained from the Korea Institute of Child Care and Education and are available at [https://panel.kicce.re.kr/pskc/index.do] with permission from the institute (accessed on 1 May 2025).
